# Associations between psychological or biological stress indicators and gut microbiota in pregnant women – findings from a prospective longitudinal study

**DOI:** 10.1186/s12866-025-04146-6

**Published:** 2025-07-19

**Authors:** Jakub Kreisinger, Šárka Kaňková, Daniela Dlouhá, Jana Ullmann, Kamila Nouzová, Hana Hrbáčková, Lucie Schmiedová, Lea Takács

**Affiliations:** 1 Department of Zoology, Faculty of Science, Charles University, Vinicna 7, Prague 2, Prague, 128 44 Czech Republic; 2 Department of Philosophy and History of Science, Faculty of Science, Charles University, Prague, Czech Republic; 3 ProfiGyn, s.r.o, Municipal Health Centre Prague, Prague, Czech Republic; 4 Department of Gynecology and Obstetrics of the First Faculty of Medicine and General Teaching Hospital, Charles University, Prague, Czech Republic; 5 Institute of Vertebrate Biology, Czech Academy of Sciences, Brno, Czech Republic; 6 Department of Psychology, Faculty of Arts, Charles University, Nám. J. Palacha 2, Prague 1, Prague, 116 38 Czech Republic; 7 Department of Gynecology and Obstetrics, the First Faculty of Medicine, Bulovka University Hospital, Charles University, Prague, Czech Republic

**Keywords:** Gut-brain axis, Microbiome, Metagenome, Dysbiosis, Prenatal stress, Depression, Anxiety, Cortisol

## Abstract

**Background:**

The perinatal period has been linked with higher vulnerability to stress and symptoms of depression and anxiety, as well as with dynamic changes in the composition of maternal gut microbiota. While recent studies indicated significant associations between stress, depression, or anxiety, and alterations in gut microbiota in pregnant women, research in this avenue is still emerging, with existing studies often being limited by small sample sizes.

**Method:**

We conducted a prospective longitudinal study of 171 women, collecting gut microbiota samples in each trimester of pregnancy and in the early postpartum, questionnaire data (perceived stress via the Perceived Stress Scale, symptoms of depression via the Edinburgh Postnatal Depression Scale, and anxiety via the 6-item State-Trait Anxiety Inventory) twice in each trimester and twice in the early postpartum period, and blood samples for cortisol levels analysis in the first and third pregnancy trimesters. Gut microbiota samples were analyzed by amplicon sequencing of 16S rRNA gene.

**Results:**

Perceived stress and symptoms of depression and anxiety showed moderate temporal changes and a high consistency at the individual level over the study period. Cortisol levels rose significantly from the first to the third trimester. There were significant temporal changes in microbiota composition between the first and second trimesters, and between the first and third trimesters. After controlling for false positive findings due to multiple testing, we found no significant associations between stress-related variables (perceived stress, cortisol levels, symptoms of depression and anxiety) and gut microbiota diversity, microbial community composition, or relative abundances of individual bacterial taxa.

**Conclusions:**

The present study results contradict previous research that indicated significant associations between emotions and gut microbiota in the perinatal period. Although we cannot provide an ultimate explanation for this discrepancy, we propose it can lie in insufficient control for false positives in the differential abundance analyses in most previous studies.

**Supplementary Information:**

The online version contains supplementary material available at 10.1186/s12866-025-04146-6.

## Background

As a period of major physiological and psychosocial changes [1–3], the perinatal period can be a time of increased stress, and is considered a window of higher vulnerability to mental health problems, particularly depression and anxiety [4]. Apart from well-studied normative physiological changes, such as changes in hormonal and immune systems [3, 5], recent research has also indicated the dynamic changes in the composition of maternal gut microbiota [6–9], a complex ecosystem comprising hundreds of species of eubacteria as well as archaea, fungi, and protists [10, 11].

Recent integration of microbiological research and neuroscience has provided increasing evidence for a bidirectional communication between the gut microbiota and the central nervous system, also referred to as the “gut-brain axis” [12]. This communication is mediated by various mechanisms, including the interaction of the microbiota with the immune system, the vagus nerve, the enteric nervous system, or the host neuroendocrine system through various bioactive substances produced by the microbiota [13]. Given the vast psychosocial changes associated with experiences of stress [4], as well as dynamic changes in gut microbiota composition in pregnant women and new mothers [6–9], the gut-brain axis interactions in the perinatal period are of great interest.

Previous research has indicated that stress in pregnancy may affect maternal microbiota composition [14, 15], however, as noted above, the signals are bidirectional, suggesting that the stress-related changes in gut microbiota composition may, in turn, affect pregnant women’s stress-reactivity and mental health [7, 16, 17] with potential cascade effects. Stress in pregnancy may exert effects on maternal gut microbiota through various pathways, including altering endocrine and immune system activity [18, 19]. At the same time, microbiota can influence maternal emotional well-being directly via the interaction with the central nervous system, or indirectly, e.g., by modulating metabolism or the immune system functioning [20]; for example, increased secretion of pro-inflammatory cytokines during pregnancy has been linked with higher levels of depressive symptoms [21, 22].

Despite the solid foundation of the “gut-brain axis” concept, our understanding of the role of microbiota in emotions and mental health in the perinatal period is far from complete and conclusive. Although there is some evidence for associations between pregnant women’s emotions or stress and gut microbiota [7, 14, 15, 23], research in this area is still emerging.

The aim of the present study was to investigate longitudinal associations between stress and gut microbiota across the perinatal period, while employing multiple indicators of stress, including perceived stress, blood cortisol levels, and symptoms of depression and anxiety. To minimize the effects of confounding factors on the correlation between the stress-related variables and microbiota, we leveraged data from a medically low-risk cohort of pregnant women with a higher educational level and socioeconomic status. Following the traditional framework for microbiota analyses, we examined whether maternal stress indicators correlate with microbial alpha diversity and composition. Since increased microbiota heterogeneity is a common hallmark of deteriorating health, likely due to impairment of host-intrinsic mechanisms regulating microbial populations [24], we also investigated a possible link between stress-related variables and microbiota heterogeneity. In addition, we used longitudinal data to investigate the patterns of temporal stability of microbiota composition and linked them with stress-related variables.

## Methods

### Procedure and participants

This study is part of a broader project focusing on perinatal factors affecting maternal and child well-being and health. Pregnant women were recruited between June 2019 and September 2022 in collaboration with three gynecological clinics in Prague, Czech Republic. The women were approached by healthcare professionals during early pregnancy check-ups (between 5th and 14th weeks) and invited to participate in the study.

After recruitment, participants provided information on their socio-demographic background. They completed self-report questionnaires measuring perceived stress, and symptoms of depression and anxiety, at eight time points: at 7.6 ± 1.2 gestational weeks (gw) (T1), 10.3 ± 1.0 gw (T2), 13.3 ± 1.4 gw (T3), 21.1 ± 1.9 gw (T4), 30.6 ± 1.4 gw (T5), 35.0 ± 1.3 gw (T6); 1.8 ± 2.8 (T7) and 7.4 ± 2.3 at (T8) weeks postpartum. Data on health status including medications and body mass index were collected in each trimester. Data on perinatal outcomes (birth weight, Apgar score) and the sex of the child were collected via questionnaires administered to the mothers in the early postpartum period. The participants provided blood samples for cortisol level analysis in the first (10.3 ± 1.0 gw) and third (32.6 ± 1.6 gw) pregnancy trimester. They provided stool samples for microbiota analysis once in each trimester (at 10.3 ± 1.4, 25.3 ± 2.4, and 33.9 ± 1.8 gw) and then again at 7.0 ± 1.9 weeks postpartum.

Inclusion criteria for this study were: fluency in Czech, no serious health problems or psychiatric treatment, no IVF. A total of 257 women were recruited. Out of those women, 18 dropped out of the study during pregnancy and 16 of them miscarried. We excluded women who did not provide stool sample (*N* = 51). After applying exclusion criteria, we had 172 women with microbiota samples available.

### Measures of psychological indicators of stress

Maternal perceived stress was assessed by the 10-item Perceived Stress Scale (PSS), as described in Cohen et al. [25], which measures how often the participants found their lives to be stressful, unpredictable, uncontrollable and overwhelming over the last two weeks. Each item is rated on a 5-point scale ranging from 0 to 4. The total score may thus range from 0 to 40, with higher scores indicating higher levels of stress. The PSS has previously been used in a population of pregnant and postpartum women [26, 27]. Cronbach’s α across the eight time points ranged from 0.82 to 0.88, indicating good reliability of the measure.

Symptoms of depression were assessed via the Edinburgh Postnatal Depression Scale (EPDS), as described in Cox et al. [28]. The EPDS is a 10-item self-report questionnaire originally developed to assess depressive symptoms in the postpartum period, however, it has also been validated for pregnant women [29]. Each item is rated on a 4-point scale ranging from 0 to 3. The total score may thus range from 0 to 30, with higher scores indicating higher levels of depressive symptoms. Cronbach’s α across the eight time points ranged from 0.81 to 0.86, indicating good reliability of the measure.

Anxiety symptoms were assessed via the 6-item short-form of the state scale of the State-Trait Anxiety Inventory (STAI), as described in Marteau and Bekker [30]. The participants indicate how they feel right now, at the given moment. Each item is rated on a 4-point scale ranging from 1 to 4. The total score may range from 6 to 24; the higher the scores, the higher the anxiety levels. Cronbach’s α across the eight time points ranged from 0.79 to 0.89, suggesting a good reliability of STAI.

### Blood cortisol analysis

Blood samples were collected from 137 women in the first and from 134 women in the third trimester of pregnancy during the medical checkups at the gynecological clinics. Blood samples were analyzed for cortisol concentrations in blood serum. Blood serum was obtained after centrifugation (2 min at 3000× g at 21◦C) and stored at − 20◦C until analyzed. The cortisol analysis was performed using the advanced gas chromatography tandem mass spectrometry (GC-MS/MS) platform. This method was validated for the blood of pregnant women and for mixed umbilical cord blood [31]. The cortisol analysis was carried out at the Institute of Endocrinology in Prague, Czech Republic.

### Microbiota profiling

Stool samples were collected by participants who were provided with a collection kit and detailed instructions on collecting and storing a stool sample. The stool samples (approx. 2 g) were collected with a plastic spoon and stored in sterile containers filled with 99.9% ethanol until they were brought to the genome laboratory, where they were stored at −20°C until DNA isolation.

Metagenomic DNA from gut tissue samples was extracted using the PowerSoil DNA Isolation Kit (Qiagen). Sequencing libraries were prepared using a two-step polymerase chain reaction (PCR) protocol as described in Čížková et al. [32]. In the first PCR, standard metabarcoding primers from [33] targeting the V3–V4 region of the bacterial 16S rRNA gene (S-D-Bact-0341-b-S-17: CCTACGGGNGGCWGCAG and S-D-Bact-0785-a-A-21: GACTACHVGGGTATCTAATCC) were used. These primers were extended by inline barcodes and priming sites for the second PCR. In the second PCR, dual indexes were introduced, and Illumina-compatible Nextera-like sequencing adapters were added. Each metabarcoding PCR was performed in technical duplicate to account for PCR and sequencing stochasticity. PCR products were pooled based on their concentration and sequenced in two runs on the MGI platform (300 bp paired-end reads) at CEITEC, Brno, Czech Republic.

After demultiplexing the sequencing data with skewer [34], the fastq files were quality filtered (maximum number of expected errors < 3) and denoised with dada2 [35] run with the parameters pool=”pseudo” to increase the sensitivity of detection of rare ASVs [36]. The resulting amplicon sequence variants (ASVs) were checked for the presence of chimeric ASVs using the uchime2 [37] software and the Silva v. 138 reference database [38]. The consistency of ASV profiles between technical duplicates was then checked using Procrustes analysis. The duplicated data were then merged, eliminating any ASVs that were not detected in both duplicates. To reduce data redundancy, ASVs were clustered into Operational Taxonomic Units (OTUs) using vsearch [39] based on 97% sequence similarity, and taxonomic assignment of their representative sequences was performed using the RDP classifier [40] and the Silva v. 138 database. OTUs that were not assigned to the phylum level or that correspond to chloroplasts and mitochondria were not included in subsequent analyses.

A total of 683 gut microbiota samples from 172 participants were collected. To eliminate possible effects of antibiotic treatment on the microbiota, we excluded microbiota samples collected in the trimester the participant reported antibiotic treatment and also those collected in the trimester following the trimester with antibiotic treatment (*n* = 27). In addition, six samples were excluded due to inconsistencies between technical replicates and one sample was excluded due to a low number of high-quality sequences. Consequently, the final dataset for the statistical analyses included 649 gut microbiota samples from 171 participants in total (170 samples from the first trimester, 166 from the second trimester, 160 from the third trimester and 153 from six to eight weeks postpartum). Microbiota data from all four time points were available for 143 women, from three time points only for 23 women, from two time points only for three women and from one time point only for two women. All microbiota data available entered the statistical analyses. The final database of microbial data consisted of 7,902,532 high-quality sequences (mean sequencing depth per sample = 12176, range = 1133–30940) corresponding to 937 OTUs.

### Statistical analyses

As a first step, we examined the longitudinal variation in stress-related variables (stress, cortisol, depression, and anxiety levels) using Linear Mixed Effect Models (LMM) fitted with the R package lme4. A study time point was included as a factorial fixed effect and individual identity as a random effect. Variance explained by the fixed effect (*R*^2^ marginal) and both the fixed and random effects (*R*^2^ conditional) was calculated following Nakagawa and Schielzeth [41] to assess the strength of temporal variation and within-subject consistency in the response variables tested. These analyses revealed that variance associated with temporal variation was much smaller compared to within-subject consistency for all psychological variables (see the Results section for details). Therefore, for analyses testing the association between these variables and gut microbiota, we used the mean scores of the psychological variables for (i) the period of pregnancy, (ii) the postpartum period, and (iii) their changes from pregnancy to the postpartum. Most stress-related variables were strongly correlated with each other (see Results section); to avoid problems with multicollinearity, their effects on the gut microbiota were examined in separate statistical models.

The analyses examining associations between stress-related variables and microbiota focused on four aspects of microbiota community structure: (i) microbiota composition, (ii) microbiota alpha diversity, (iii) temporal stability, and (iv) microbiota heterogeneity (i.e., individual-level dispersion).

Variation in microbiota composition was analyzed using linear decomposition models fitted with the R package LDM [42]. The main advantage of this approach compared to the existing alternatives is that analyses at the whole community level (i.e., beta diversity analyses analogous to PERMANOVA or redundancy analyses) and the OTU level (i.e., differential abundance) are performed in a single step, reducing the risk of discrepancies often reported in the literature. Using LDM, we first tested the differences in microbiota composition among study time points using the entire dataset. To account for repeated sampling of the same individuals, individual identity was considered as a constraint (i.e., “strata”) for the LDM permutations. However, this approach is not applicable for analyses testing the effects of stress-related variables, as these are invariant at the individual level. Therefore, separate analyses were performed for the microbial data from each study time point.

Variation in microbial alpha diversity was analyzed using the Shannon diversity index and OTU richness (i.e., the total number of OTUs detected in each sample), calculated using the rarefied OTU table (rarefaction threshold = 1133 reads per sample).

To analyze temporal stability, Bray-Curtis dissimilarities (based on the relative abundance of OTUs) and Jaccard dissimilarities (based on the absence/presence of OTUs) were calculated. Subsequently, dissimilarity values for samples corresponding to different time points but the same individual were extracted and used in statistical analyses.

PERMDISP (i.e., the betadisper function from the R package vegan) was used to quantify microbiota heterogeneity. For each sample, we calculated the Euclidean distance to an ordination centroid, which was defined separately for each study time point. The association between stress-related variables and microbiota alpha diversity, individual-level stability, and microbiota heterogeneity was tested using general linear models.

Preliminary analyses indicated moderate but significant variation in microbiota profiles between the two sequencing runs. Therefore, all statistical models were adjusted for the variation between the sequencing runs. In addition, we examined the possible effects of maternal age, parity, gestational diabetes, pre-pregnancy BMI, the difference between pre-pregnancy BMI and second-trimester BMI, and infant sex on the alpha diversity and composition of microbiota using LDM. Variables with a significant effect were included as covariates in analyses examining the effect of stress-related variables on gut microbiota.

As the stress-related variables showed strong positive correlations, a separate model was created for each of them, which inevitably leads to an inflation of false-positive results. To account for the false discovery rate, we corrected the resulting p-values for multiple testing using the method of Benjamini and Hochberg [43], and only results with false discovery rates (FDR) < 0.05 were considered significant.

## Results

### Sample characteristics

The sample consisted of 171 women with a mean age of 31.5 years (s.d. = 4.1) years. Out of those women, 63.9% were primiparous, 78.6% had a university degree and 55% were married. The prevalence of hypertension and gestational diabetes was 4.1% and 11.7%, respectively. The average body mass index before pregnancy was 23.1 ± 3.8. A total of 76.6% of the participants gave birth vaginally, and 56.8% of women gave birth to a girl. The mean birth weight was 3404 ± 430 g and the mean Apgar score at 5 min was 9.71 ± 0.64. The sample characteristics are shown in Table 1 and in Table S1 (Additional file 1).

**Table 1:** Participants’ characteristics

Maternal age, years, mean (SD)	31.5 (4.1)
Maternal age, years, range	23–44
Parity, n (%)	
Primipara	108 (63.9)
Multipara	61 (36.1)
Maternal education, n (%)	
Elementary	1 (0.6)
Secondary	35 (20.7)
University	133 (78.6)
Marital status, n (%)	
Single	75 (44.4)
Married	93 (55.0)
Divorced	1 (0.6)
Child sex, n (%)	
Girl	96 (56.8)
Boy	73 (43.2)
Monthly family income, CZK, n (%)	
<15,000	0 (0%)
16,000–30,000	1 (0.6%)
31,000–45,000	23 (13.7%)
46,000–60,000	46 (27.4%)
61,000–75,000	42 (25.0%)
76,000–90,000	29 (17.3%)
>91,000	27 (16.1%)
Maternal health status in pregnancy	
Hypertension, n (%)	7 (4.1)
Diabetes, n (%)	20 (11.7)
BMI (prepregnancy), mean (SD)	23.1 (3.8)
BMI (2nd trimester), mean (SD)	24.8 (3.8)
Delivery mode, n (%)	
Vaginal	128 (76.6)
Planned caesarean section	21 (1.6)
Emergency caesarean section	15 (9.0)
Operative vaginal delivery	3 (1.8)
Birth weight, grams, mean (SD)	3,404 (430)
Birth weight, grams, range	2,120–4,610
Gestational age at birth, days, mean (SD)	278 (8.7)
Gestational age at birth, days, range	244–291
Apgar score at 5 min, mean (SD)	9.7 (0.6)
Apgar score at 5 min, range	8–10

The number of participants with the EPDS score of 11 or higher, a recommended cut-off for a risk of major depression [44], was 21 (13.6% of participants who completed the questionnaire) at T1, 20 (12.0%) at T2, 19 (12.8%) at T3, 12 (7.3%) at T4, 13 (7.7%) at T5, 22 (13.2%) at T6, 28 (17.2%) at T7, and 27 (17.0%) at T8.

### Temporal changes in stress-related variables and gut microbiota

#### Temporal changes in perceived stress, symptoms of depression and anxiety, and cortisol levels

According to LMM and Tukey post-hoc comparisons, perceived stress was constant during pregnancy, with a non-significant increase at two weeks postpartum, and a significant increase at six to eight weeks postpartum (Additional file 2, Figure S1). Importantly, temporal changes in perceived stress were small (i.e., *R*^2^ marginal = 1.8%) compared to 62% of the variance explained by the combination of individual identity and temporal changes (i.e., *R*^2^ conditional).

There were no large temporal changes in depressive symptom levels during the study period (Additional file 2, Figure S1). Temporal changes in depressive symptoms explained only 0.8% of the variation in depressive symptom scores, whereas the effect of individual identity explained 55.5% of the total variation in depressive symptom scores.

Anxiety levels showed a non-significant decreasing trend during pregnancy, followed by a significant increase at two weeks postpartum and leveling off to the values typical of late pregnancy at six to eight weeks postpartum (Additional file 2, Figure S1). However, the effect of temporal changes explained much less variation (2.5%) in anxiety scores compared to the combined effect of temporal changes and individual identity (41.9%).

As the stress-related psychological variables (perceived stress, symptoms of depression, and anxiety) showed only moderate temporal changes and, at the same time, a high consistency at the individual level, we used individual-level means for each psychological variable in subsequent analyses. We calculated individual-level means based on the values for (i) all time points during pregnancy and (ii) the two postpartum time points. In addition, we calculated (iii) the difference between the mean values for pregnancy and the postpartum period.

Blood cortisol levels were lower in the first trimester (LMM estimate [± S.E.]: 422.68 ± 26.69) compared to the third trimester (LMM estimate: 712.51 ± 26.93, ΔD.f. =1, χ^2^ = 80.59, *p* < 0.0001). The combined effect of temporal change and individual identity explained 56.14% of the variation in cortisol levels, while the temporal difference in cortisol levels explained 17.62% of the variation in cortisol levels.

#### Temporal changes in microbiota alpha diversity and composition

LMMs showed no significant effect of the study time point on alpha diversity (Additional file 1, Table S2). We also found no significant effects of potential covariates (maternal age, parity, gestational diabetes, pre-pregnancy BMI, a change between pre-pregnancy BMI and second-trimester BMI, and infant sex) on alpha diversity, but we did detect an effect of the sequencing run and sequencing coverage (Additional file 1, Table S2).

**Fig. 1:**
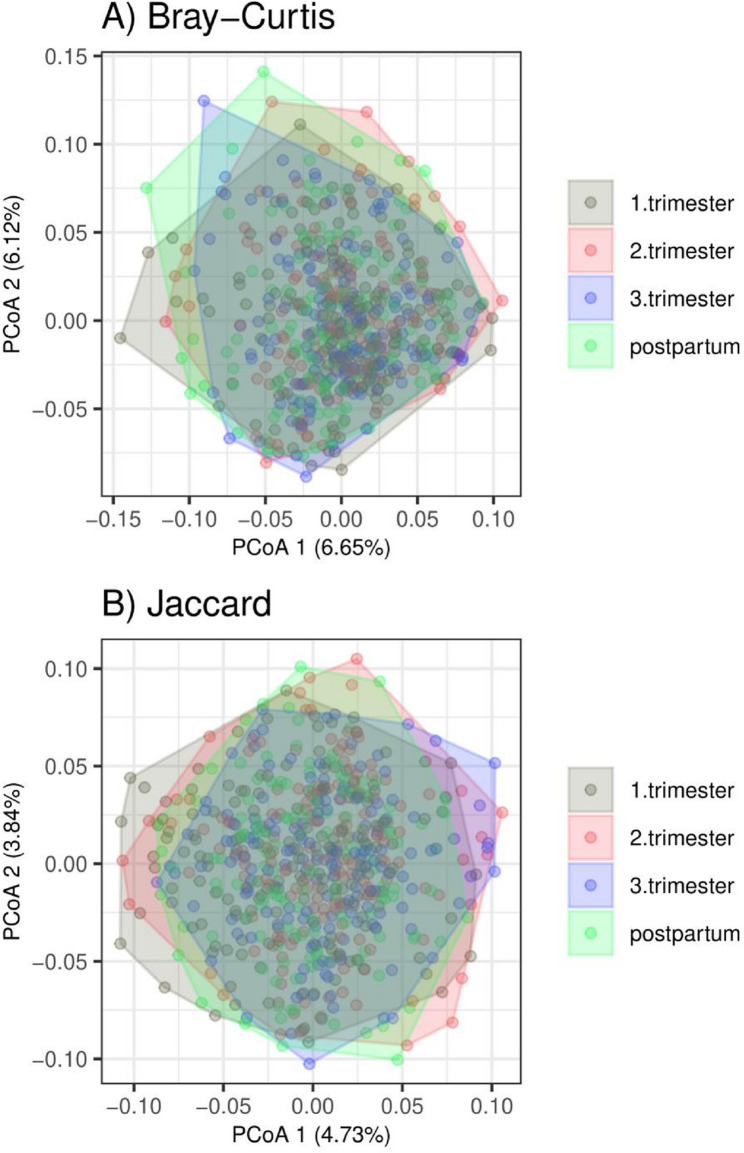
Principal coordinate analyses showing variation in microbiota composition between study time points based on Bray-Curtis and Jaccard dissimilarities. Data from different phases of the perinatal period are indicated by different colors and Hull-convex polygons. Variation between sequencing runs was statistically removed using the LDM::adjust.data.by.covariates() function

**Fig. 2:**
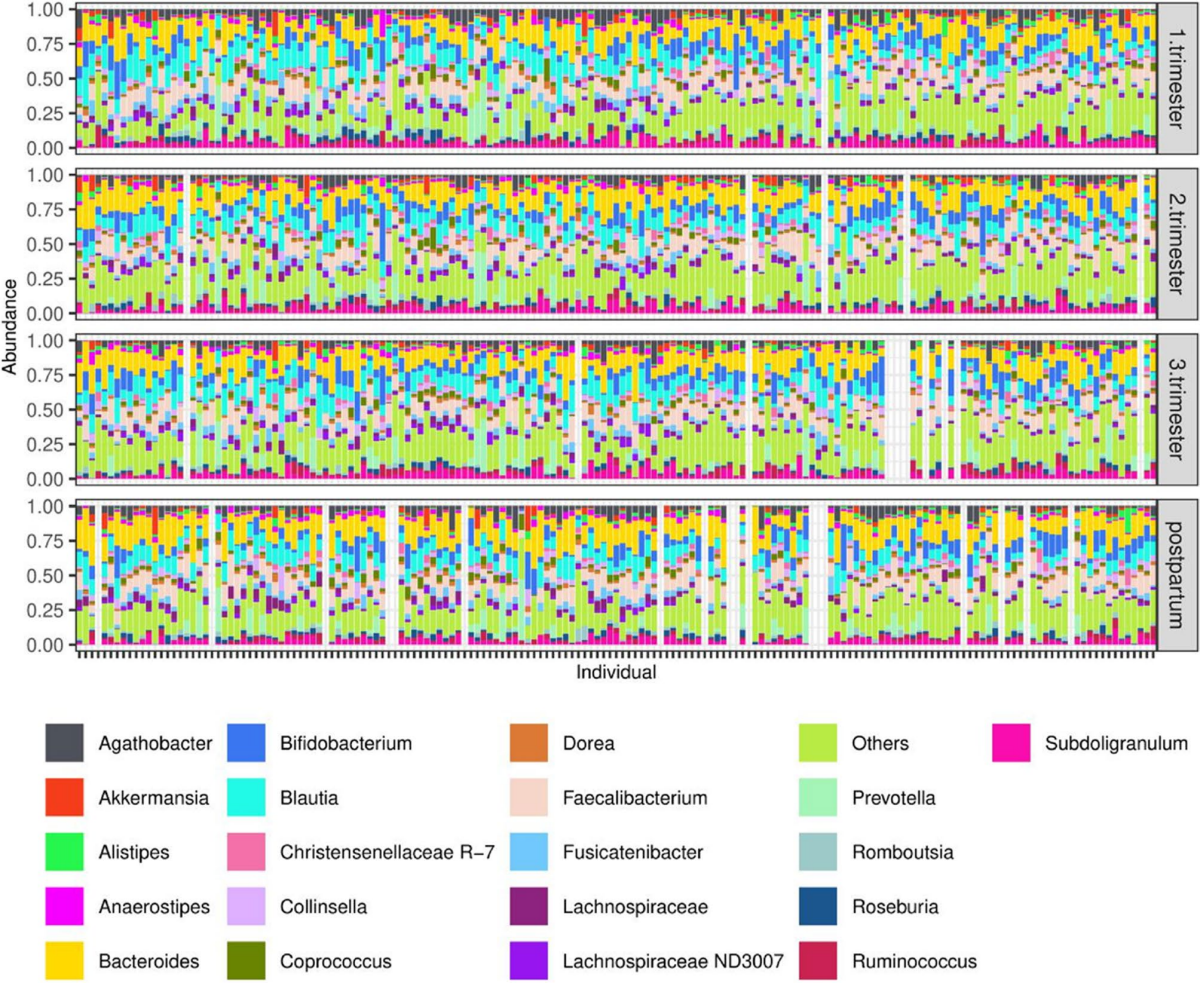
Relative abundance of the dominant bacterial genera or higher bacterial taxa detected at each study time point

Exploratory analysis based on PCoA (which was adjusted for sequencing run identity, Fig. 1), similar to taxonomic bar charts (Fig. 2), showed no pronounced differences in microbiota composition at different study time points. However, according to LDMs including data from all study time points, there were highly significant differences in microbiota composition (*p* = 0.0001) across the study period. Separate LDMs for all pairwise combinations of the study time points revealed significant differences in microbiota composition between all study time points except for the second and third trimesters (Additional file 1, Table S3).

**Fig. 3:**
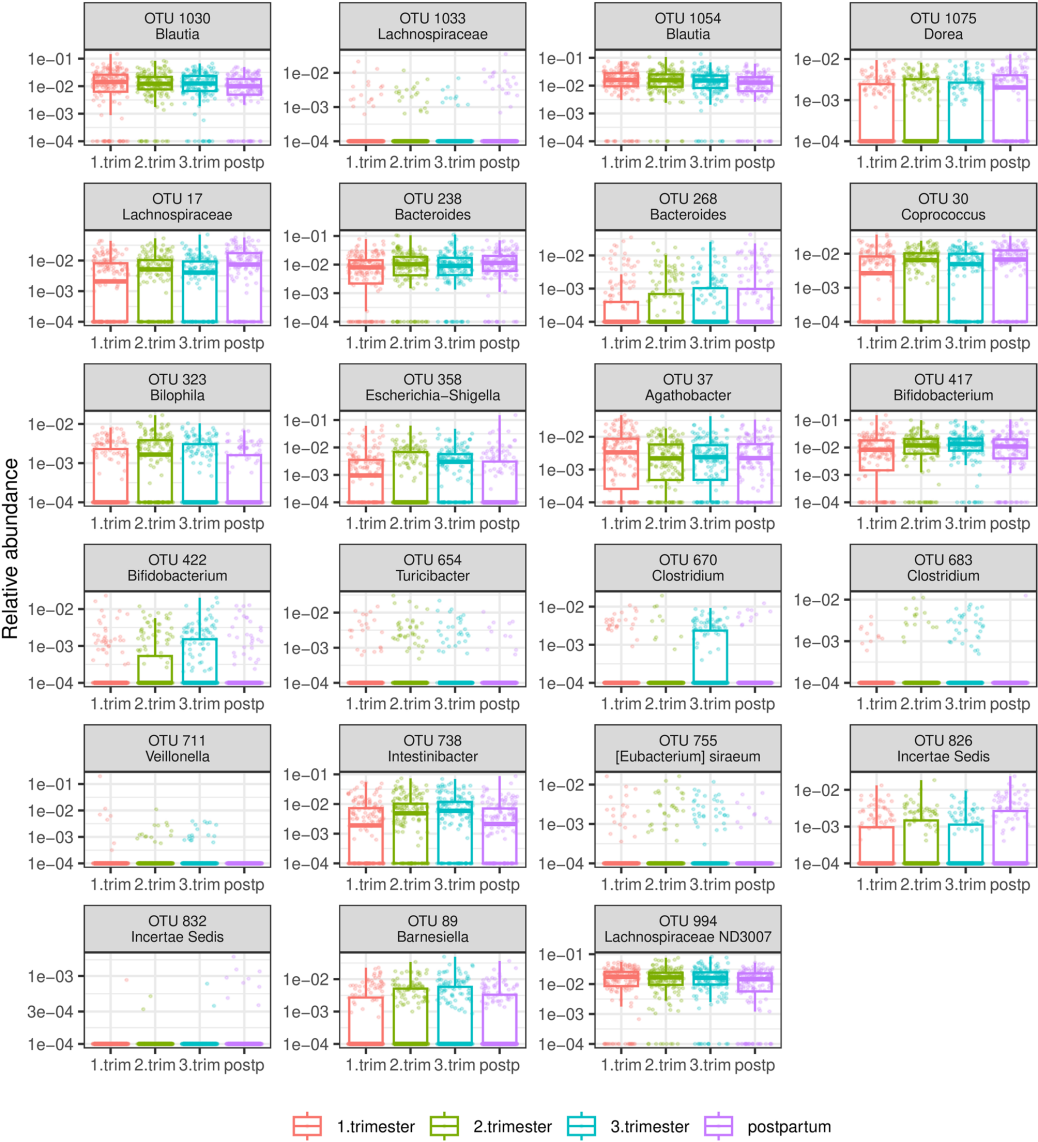
Boxplots showing the pattern of abundance variation for bacterial OTUs that exhibit significantly different abundance between study time points

A total of 23 OTUs showed significant temporal changes (Fig. 3). A subset of OTUs from the genera *Intestinibacter*, *Bilophilla*, *Bifidobacterium*, and *Barnesiella* were more abundant in the later pregnancy stages, while in the postpartum period, their abundance decreased to a level comparable to that of the first trimester. For OTUs of the genera *Coprococcus*, *Bacteroides*, and Lachnospiraceae, the increase in abundance between the first and second trimesters persisted after delivery. Finally, in OTUs of the genera *Blautia* and *Agathobacter*, a gradual decline was observed throughout the perinatal period. Lachnospiraceae ND3007 remained constant during pregnancy and showed a specific decline in the postpartum period.

LDMs fitted separately for each study time point showed significant effects of pre-pregnancy BMI on microbiota composition in the first trimester, and of parity on gut microbiota composition in the second trimester (Additional file 1, Table S4). However, these analyses revealed no significant association between these covariates and specific OTUs.

### Correlations between stress-related variables

The pairwise correlations between all stress-related variables are shown in Additional file 2, Figure S2. In general, most of the psychological variables showed strong positive mutual correlations for pregnancy and the postpartum period, while a significant correlation between cortisol levels and psychological variables was found only in a few cases.

### Associations between stress-related variables and gut microbiota

#### Associations between stress-related variables and gut microbiota diversity and composition

The LMM showed no association between the stress-related variables (perceived stress, symptoms of depression and anxiety, and cortisol levels) and microbial alpha diversity at any study time point (Additional file 1, Table S5). Interactions with the study time point were not significant after the multiple testing correction, suggesting that the effect of stress-related variables on alpha diversity did not vary during the study period.

LDM analyses correlating the microbiota composition at all study time points with stress-related variables from the pregnancy period, the postpartum period, or with their changes from pregnancy to the postpartum (considering only the sequencing run as a covariate) showed a significant association between microbiota composition in the first trimester and mean levels of depressive symptoms and perceived stress in pregnancy, and cortisol levels in the first trimester. All the effects remained significant in the models that controlled for sequencing run, pre-pregnancy BMI, and parity. In addition, adjusted models also showed an association between mean pregnancy anxiety levels and microbiota composition in the first trimester. Importantly, the significance of all these effects was relatively weak and disappeared after the correction for multiple testing (Additional file 1, Table S6). The LDM analyses also did not reveal any OTU associated specifically with the stress-related variables which were found to be linked with microbiota composition prior to the multiple testing correction.

#### Associations between stress-related variables and stability in the gut microbiota composition

Although the most pronounced changes in microbiota composition at the population level occurred between the first and second trimester, at the individual level, the most dramatic changes occurred between the third trimester and the postpartum period. At the same time, microbiota showed the highest degree of within-individual stability between the second and third trimester of pregnancy (Additional file 2, Figure S3). Stress-related variables (at all study time points) showed no or only a weak association with temporal stability in microbiota composition (Additional file 1, Table S7). This effect was consistent for models adjusted for the sequencing run, pre-pregnancy BMI, and parity, or only for the identity of the sequencing run. The strongest association was found for microbiota composition changes from the second to the third trimester and changes in anxiety levels from pregnancy to the postpartum period (Bray Curtis: *p* = 0.0130; Jaccard: *p* = 0.006). Importantly, none of those results were significant after the correction for multiple testing.

#### Associations between stress-related variables and the microbiota heterogeneity

Microbiota heterogeneity, assessed based on Jaccard dissimilarities, was significantly higher in the first trimester compared to other study time points (ANOVA: F_3,645_ = 3.3568, *p* = 0.0186), but no differences were found between the study time points on the basis of Bray-Curtis dissimilarities (ANOVA F_3,645_ = 1.952, *p* = 0.1200, Additional file 2, Figure S4). We found no associations between microbiota dispersion and stress-related variables (Additional file 1, Table S8).

## Discussion

The perinatal period is characterized by vast changes in phenotype that include alterations in physiology, immune system, or emotions [1–3]. Previous studies have indicated a significant remodeling of the symbiotic gut microbiota occurring during the perinatal period and have attempted to link this variation to concomitant changes in other phenotype characteristics [6, 45, 46]. In contrast to most previous studies on gut microbiota during pregnancy, our longitudinally collected data span not only all three pregnancy trimesters, but also the early postpartum period.

Consistent with some previous reports [8, 9], we observed no variation in alpha diversity across the study period. However, some studies have reported a decrease in alpha diversity in later pregnancy stages [6] or a varying direction of change for different alpha diversity indices [47]. In line with our findings, the majority of previous studies have reported significant changes in microbiota composition during the perinatal period [6, 8, 9]. However, some studies did not observe such compositional changes [48, 49]. On the population level, we observed that the most pronounced changes in microbiota composition occurred between the first and second trimester, whereas no difference in microbiota composition was detected between the second and third trimester. In the postpartum period, the composition of microbiota did not fully converge to the microbiota typical of early pregnancy. Leveraging longitudinally collected data, we also examined the patterns of temporal stability in microbiota composition and found that the most dramatic changes at the individual level occurred between the third trimester and the postpartum period, which is not entirely consistent with the patterns of sample-level changes described above. These seemingly contradictory results might be due to the stochastic nature of the individual-level microbiota changes, which resulted in only modest systematic variation in the community composition between the third trimester and the postpartum period.

After multiple testing corrections, our data showed no association between stress-related variables and microbiota in the perinatal period. This finding is not consistent with prior studies, most of which were based on smaller sample sizes than our research and reported significant associations between microbiota and perceived stress [7, 15, 16], depressive symptoms [15, 17, 50, 51], or anxiety [50] in the perinatal period. More generally, studies outside the perinatal period provided important evidence for the role of microbiota in mental health by showing that symptoms of psychiatric disorders such as schizophrenia [52], major depression [53, 54] or anxiety [55] can be partially transferred from humans to animal models by fecal microbiota transplantation. Nevertheless, while previous smaller-sample studies reported significant findings in terms of the association between stress or emotions and gut microbiota in pregnancy, recent research based on large population samples suggests that this association is weak (less than 0.2% of explained variation) [56, 57]. Importantly, a recent systematic review pointed out that the evidence for the association between anxiety or depressive symptoms on the one hand, and the alpha diversity or community composition of gut microbiota or the abundance variation of specific microbial taxa on the other, is not entirely consistent [58].

There may be many reasons for the discrepancy between our results and those of previous studies that found significant associations between stress or emotions and gut microbiota in the perinatal period. Since previous studies included data from multiple ethnic groups from three continents, it seems unlikely that we were unable to detect this association due to specific characteristics of our study cohort. Furthermore, we can rule out that we were not able to detect this association due to the heterogeneity of our study cohort; in fact, our sample consisted of healthy, low-risk women with a generally high educational level and socioeconomic status. It is however possible that we found no effects of stress because of the small variation in stress and related emotions in our cohort. Nevertheless, similar to our study, previous studies reporting significant associations between emotions and microbiota in pregnancy were based on low-risk, healthy samples with a relatively moderate variation in levels of emotions, where women suffering from psychiatric problems were excluded from the analyses [17, 50]. Additionally, we cannot rule out the possibility that the association between microbiota and stress-related variables in our sample was masked by unmeasured confounding factors, such as maternal diet, probiotic use, physical activity, sleep quality, or social support.

The discrepancy between our results and previous work may also be at least partly related to the overall weak association between stress-related variables and gut microbiota in the normal population [56, 57], meaning that the probability of detecting the association in a population sample of tens to hundreds of individuals is relatively low. Importantly, the significant findings of previous studies may partly result from insufficient control for false positive discoveries. Most previous studies failed to detect the effects of maternal stress or emotions on variation in microbiota composition at the whole community level, i.e., beta diversity [15–17], or the effects were at the edge of statistical significance [14, 51], or the beta diversity analyses did not properly control for multiple data time points from the same individual [50]. As a result, the conclusion that there is a correlation between stress or emotions and gut microbiota composition was mainly based on the results of differential abundance analyses aimed at identifying specific taxonomic traits that are correlated with stress or emotions. However, most of the existing tools used for differential abundance analyses do not hold false positives at the nominal level, and the results provided by different tools for differential abundance analyses are highly inconsistent [59]. It is therefore strongly recommended that the results of differential abundance analyses should be interpreted with caution and not separately from the results of beta diversity analyses designed to test associations at the whole community level. In addition, statistical approaches that simultaneously infer variation in beta diversity and differential abundance, such as the LDM package used in our study [42] or the mvabund software used in community ecology [60], may be helpful in mitigating discrepancies in the results at these two closely related levels of microbiota complexity.

## Conclusions

Observations in various empirical science fields have shown that effect sizes decrease and negative results increase with more recent publication dates [61, 62]. In line with this pattern, our study contradicts earlier reports of a significant correlation between stress-related factors and microbiota during the perinatal period. Our longitudinal study, involving multiple data collection time points, a large sample size, and a homogeneous group of healthy, low-risk women, found no association between stress and microbiota in the perinatal population. This discrepancy may be due to the small effect size of stress-related factors on microbiota in a healthy population and the inadequacy of analytical tools used in most previous studies in terms of controlling for false positives. These issues can lead to serious bias in our understanding of the gut microbiota’s role in human health. To mitigate these issues, researchers should share their primary data for meta-analyses, pre-register their research, and be encouraged to publish negative results [63].

## Supplementary Information

Supplementary Material 1.

Supplementary Material 2.

## Data Availability

Sequencing data associated with this project are archived in the European Nucleotide Archive (project accession number: PRJEB85512). Accession numbers for each sample are provided in Additional file 1,Table S1.

## Abbreviations

gw: Gestational weeks
IVF: In vitro fertilization
PSS: Perceived Stress Scale
EPDS: Edinburgh Postnatal Depression Scale
STAI: State-Trait Anxiety Inventory
GC MS/MS: Gas chromatography tandem mass spectrometry
DNA: Deoxyribonucleic acid
PCR: Polymerase chain reaction
rRNA: Ribosomal ribonucleic acid
ASV: Amplicon sequence variant
OTU: Operational Taxonomic Unit
LMM: Linear mixed effect model
LDM: Linear decomposition model
PERMANOVA: Permutational multivariate analysis of variance
BMI: Body mass index
FDR: False discovery rates
ANOVA: Analysis of Variance
