# Supplementary material for: Associations between psychological or biological stress indicators and gut microbiota in pregnant women – findings from a prospective longitudinal study
Source: BMC Microbiol. 2025 Jul 19;25:442. doi: 10.1186/s12866-025-04146-6 (PMC12275315; doi:10.1186/s12866-025-04146-6)
Supplement: Supplementary file 2 — Supplementary Material 2. [file 12866_2025_4146_MOESM2_ESM.pdf]

Associations between psychological or biological stress indicators and gut microbiota in pregnant women – findings from a prospective longitudinal study

Additional file 2: Supplementary tables S1-S8.

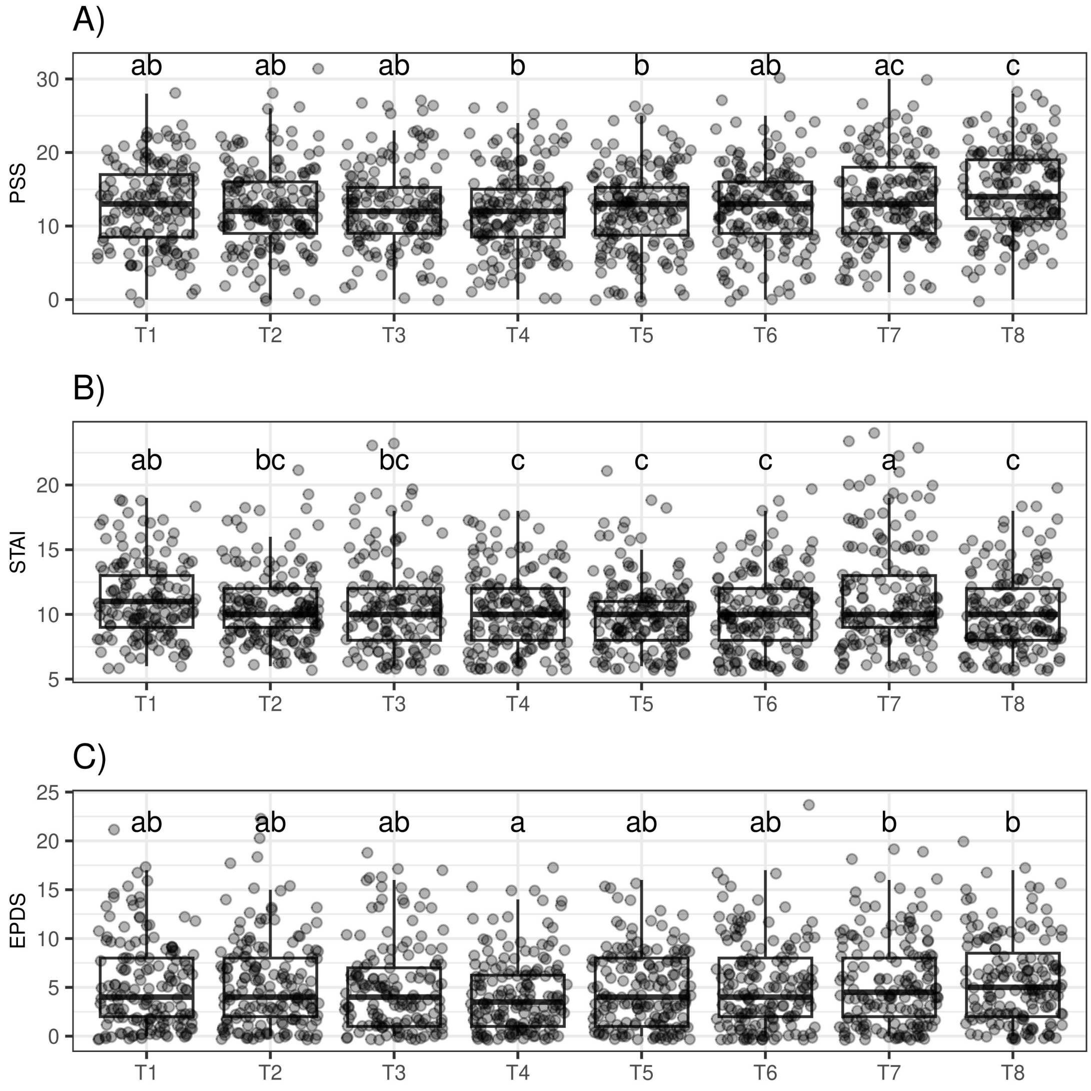

Figure S1: Boxplots showing the changes in A) perceived stress (PSS), B) anxiety (STAI) and C) depressive symptoms (EPDS) during the eight study time points: T1 and T2: 1st trimester, T3 and T4: 2nd trimester, T5 and T6: 3rd trimester, T7 and T8: postpartum period. Differences between the study time points were tested with Tukey post hoc comparisons. The scores for two time points differ significantly if the letters above the corresponding boxplots do not overlap.

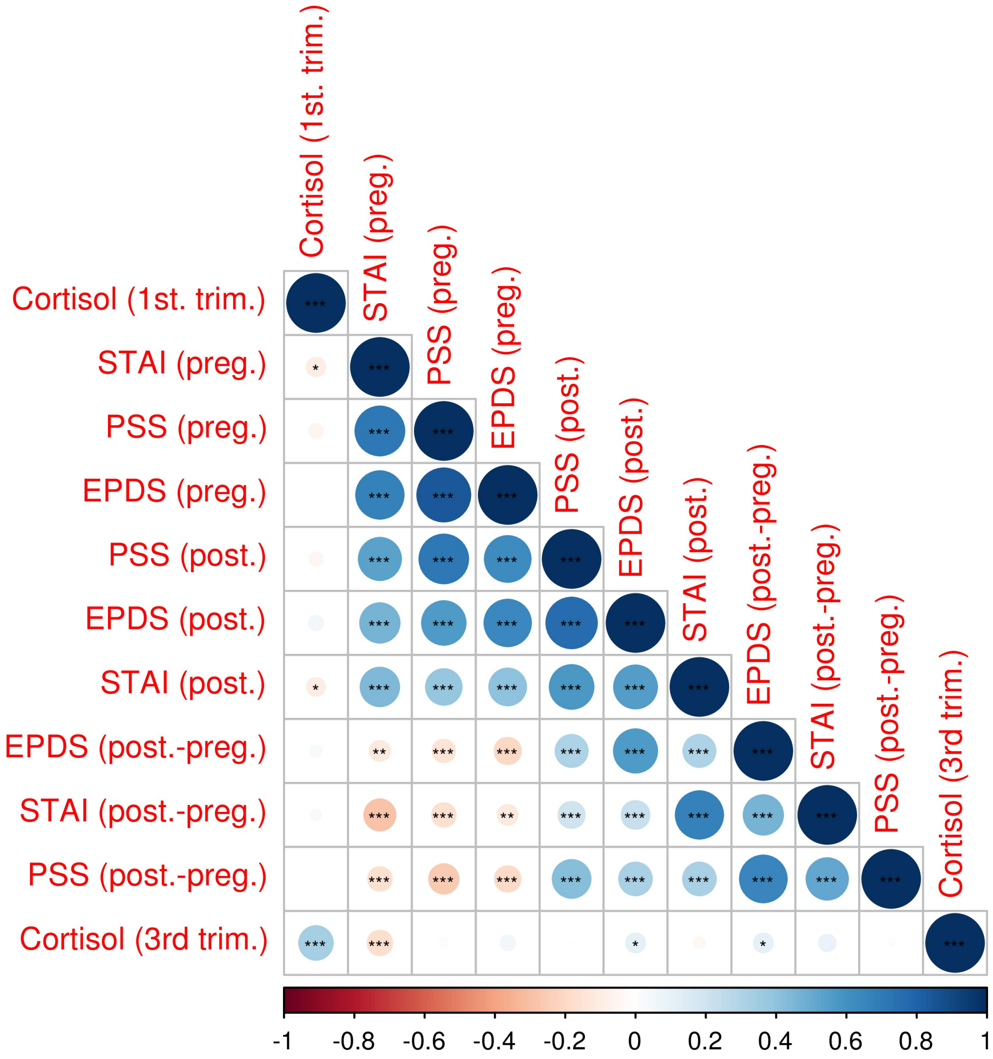

Figure S2: Heatmap of pairwise Spearman correlations between stress-related variables used in microbiota analyses: first and third trimester cortisol levels and mean PSS, EPDS and STAI scores in pregnancy (preg.), postpartum (post.) and their difference between pregnancy and postpartum (preg. - post.). Statistical significance after corrections by multiple testing is indicated by asterisks (* ~ p < 0.05, ** ~ p < 0.01 and *** p < 0.001).

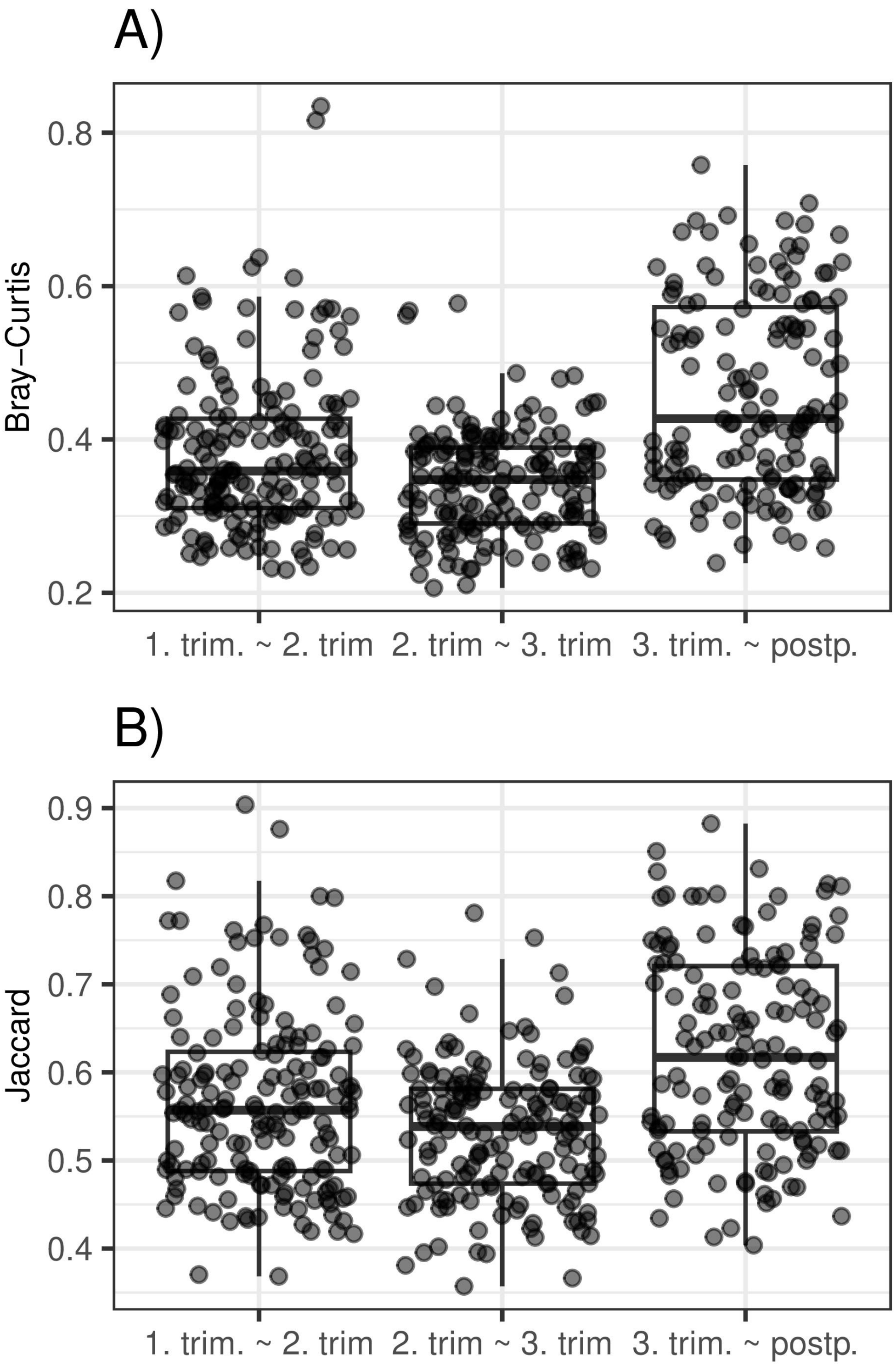

Figure S3: Within-individual chances of gut microbiota composition between successive study time points. Boxplots show changes in composition expressed as A) Bray-Curtis and B) Jaccard dissimilates between the gut microbiota of the same individual collected in the first and second trimester (1. trim ~ 2. trim), second and third trimester (2. trim ~ 3. trim), or third trimester and early postpartum period (3. trim ~ postp).

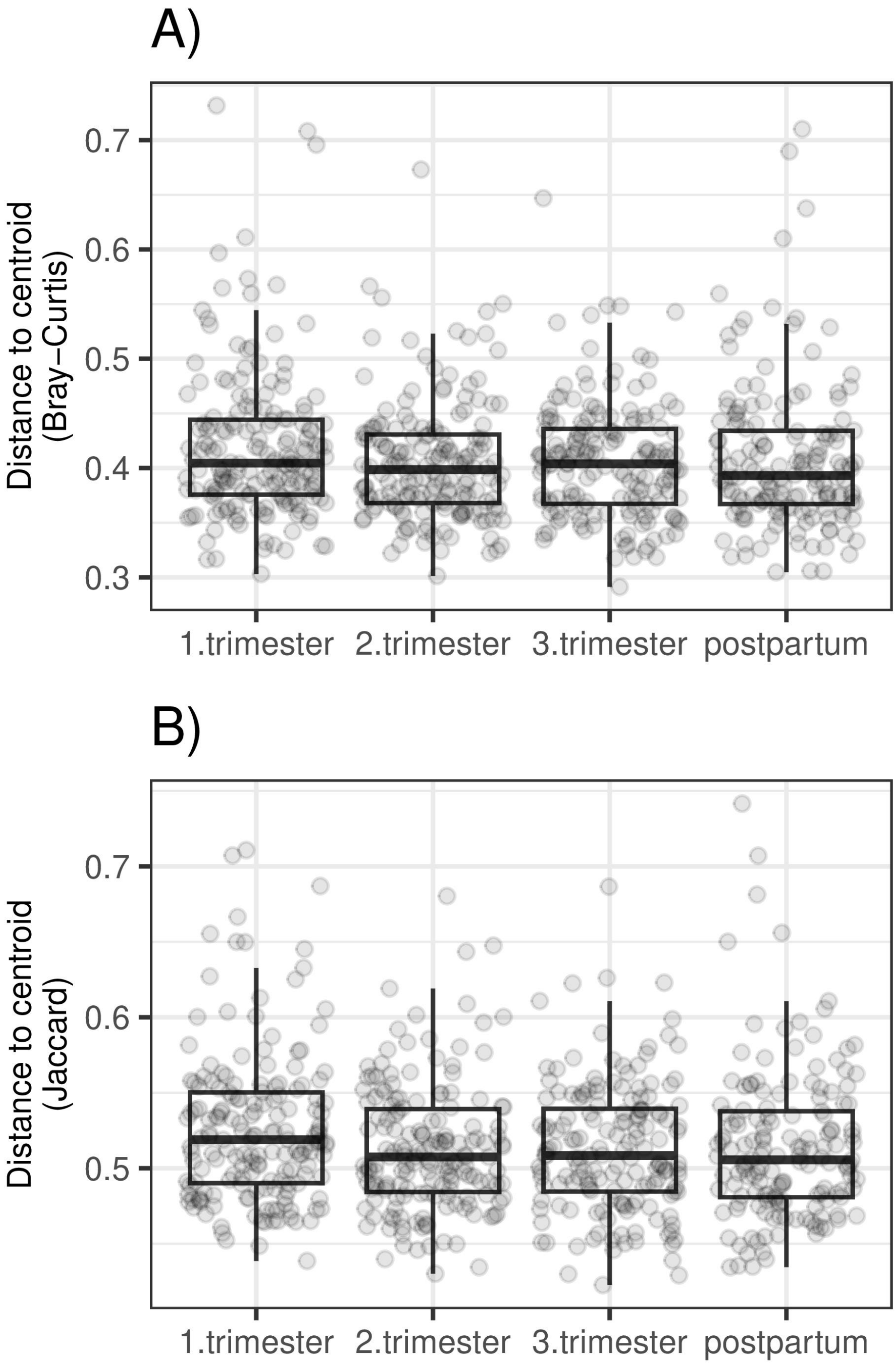

Figure S4: Heterogeneity of the gut microbiota during the study periods, expressed as Euclidean distance to the PCoA centroids for A) Bray-Curtis and B) Jaccard dissimilarities.
